# Trends in the Incidence and Survival Outcomes in Patients With Small Cell Lung Cancer in the United States: An Analysis of the SEER Database

**DOI:** 10.1002/cam4.70608

**Published:** 2025-02-05

**Authors:** Dipesh Uprety, Randell Seaton, Abesh Niroula, Tarik Hadid, Kaushal Parikh, Julie J. Ruterbusch

**Affiliations:** ^1^ Department of Medical Oncology Barbara Ann Karmanos Cancer Institute, Wayne State University Detroit Michigan USA; ^2^ Population Studies and Disparities Research Program Barbara Ann Karmanos Cancer Institute, Wayne State University Detroit Michigan USA; ^3^ Winship Cancer Institute, Emory University Atlanta Georgia USA; ^4^ Mayo Clinic Rochester Minnesota USA

**Keywords:** epidemiology, lung cancer, population, SCLC, SEER, small‐cell

## Abstract

**Background:**

There has been a lack of updated epidemiological data on the incidence and survival outcomes for patients with small cell lung cancer (SCLC) in the United States over the last two decades.

**Methods:**

A retrospective, population‐based study was conducted utilizing the Surveillance, Epidemiology, and End Results (SEER) program to identify patients with SCLC from 2000 to 2020. Trends in cancer incidence, incidence‐based mortality rates, 1‐year relative survival rates and 1‐year observed survival were evaluated utilizing the SEER database.

**Results:**

The database identified a total of 188,426 SCLC patients during the study period from 2000 through 2020. The age‐adjusted incidence rate slowly declined, on average, by 3% (95% CI: −3.2% to −2.8%) each year from 9 per 100,000 in 2000 to 4.6 per 100,000 in 2020. The decline is evident for all age groups, sexes, and races. Incidence‐based mortality also declined slowly from 6.6 in 2005 to 3.5 in 2020. However, survival outcomes, including 1‐year relative survival and 1‐year observed survivals, have not improved significantly over the last two decades.

**Conclusion:**

This study found that the incidence of SCLC has decreased from 2000 to 2020, likely due to a reduction in smoking rates, underscoring the importance of smoking abstinence. An improvement in incidence‐based mortality is likely related to an enhanced medical care and a decrease in the incidence of SCLC, but the lack of improvement in survival outcomes reflects the need for more effective systemic therapy.

## 1 Background

Small cell lung cancer (SCLC) is a subset of lung cancer with aggressive disease biology and is characterized by rapid cell proliferation. Most patients present with metastatic disease at the time of diagnosis. Cigarette smoking is the leading risk factor for SCLC. The proportion of SCLC decreased from 17% in 1986 to 13% in 2002, likely due to reduced smoking rates and changes in cigarette composition [1]. For decades, platinum‐etoposide chemotherapy has been the mainstay of the treatment. While dramatic responses are often achieved initially with chemotherapy, most patients eventually develop disease progression and succumb to the disease [2, 3]. More recently, incorporating immune checkpoint inhibitors (ICIs) with chemotherapy has improved survival in patients with extensive‐stage SCLC. Atezolizumab and Durvalumab are the two ICIs approved by the FDA on March 19, 2019, and March 27, 2020, respectively. The approvals were based on the results of the IMPOWER‐133 and CASPIAN studies [4, 5]. These agents induce a durable survival benefit in a small subset of patients. Hence, platinum‐doublet with ICIs is now the standard treatment for patients with extensive‐stage SCLC. Additionally, consolidation durvalumab after chemoradiotherapy has been recently shown to improve survival in patients with limited‐stage SCLC [6]. Moreover, advancements in radiation techniques, early‐stage at diagnosis with screening CT scans, may have improved survival for patients with limited‐stage disease. Population‐based studies to assess the survival benefit of improved cancer care, enhanced radiation techniques and the introduction of immunotherapy are lacking. Furthermore, epidemiological data on the incidence of SCLC in the last two decades have not been updated. In this study, we aim to evaluate the incidence and survival outcomes in patients with SCLC in the United States over the last two decades, utilizing the Surveillance, Epidemiology, and End Results (SEER) database.

## 2 Methods

### 2.1 Data Sources

We utilized data from the National Cancer Institute's SEER Database, which covers approximately 48% of the US population [7]. It is a reliable resource on cancer incidence and survival in the United States. We utilized data from the SEER 22 registries (excluding IL and MA) based on the November 2022 submission.

### 2.2 Study Population

We included patients diagnosed with SCLC between 2000 and 2020 for this study. International Classification of Diseases for Oncology, 3rd Edition (ICD‐O‐3) was utilized to identify patients with SCLC with ICD‐O‐3 codes of 8002, 8041–8045 [8]. We included only patients with malignant behavior and who were actively followed and had a known age. We excluded patients without survival data and whose diagnosis was made from a death certificate or at autopsy.

### 2.3 Study Measures

*Age at cancer diagnosis* was categorized into an age, in years, of less than 50, 50–64, 65–79, and 80 or older. *Age at death* mirrored this categorization. *Race and ethnicity* were combined to create a six‐level variable that represents Hispanic ethnicity inclusive of all races, non‐Hispanic White, Black, American Indian/Alaskan native, Asian pacific islander, and unknown race. Race and ethnicity were seen as nonbiological social constructs.

*SEER stage* was used to describe disease extent and spread, standardized across all cancer types where local stage describes cancer limited to initial site, regional stage describes cancer spread to lymph nodes or the surrounding tissue/organ, and distant stage describes cancer spread to distant parts to the body. SEER stage was available from 2004 to 2020 and malignant in situ cancer cases were conflated with localized stage.

*Relative survival* is a measure used to assess cancer survival by removing the impact associated with competing causes of death by relating the overall survival (OS) of cancer survivors to the expected survival in a similar cancer‐free population. Relative survival was calculated as the observed survival in the SCLC population divided by the expected survival of the general, cancer‐free population in SEER catchment areas matched on age, sex, race, region, and calendar year [9].

*Incidence‐based mortality* (*IBM*) is a method used to estimate and partition mortality rates by characteristics at time of cancer diagnosis such as age, stage, and histology. Death certificates do not provide information on cancer subtypes precluding report of death certificate mortality rates by disease subtype. SEER registries routinely link incident cancer cases with death certificate data allowing possibility of the IBM approach. By linking cause of death to incident lung cancer diagnosis, that is, IBM, we get a more accurate measurement of mortality by minimizing misattribution of lung cancer deaths and only measure true SCLC‐related mortality. IBM rates were estimated by calculating the number of lung and bronchus cancer deaths within a specific year among incident SCLC diagnoses in the numerator and the general population in SEER catchment areas of the same year in the denominator. At least 5 years of incident SCLC diagnoses prior to the first mortality year reported was used to accumulate a sufficient amount of deaths ensuring reliable IBM rates—this is referred to as a “burn‐in” period. Because our study began in the year 2000, IBM rates were reported starting as early as 2005. Rapidly fatal cancers require short burn‐in periods while cancers with longer survival times require more years of incident diagnoses to capture all possible cancer‐related deaths. In addition to establishing a “burn‐in” period before the first mortality year reported, a 5‐year follow back period of SCLC incident diagnoses prior to each additional mortality year was established to reduce long‐term survivor bias in later mortality year IBM rate estimates. For example, the numerator of IBM rates reported in 2006 and 2020 were accumulated deaths from incident diagnoses from 2001 to 2006 and 2015 to 2020, respectively [10, 11, 12].

### 2.4 Statistical Analysis

Statistical Analysis Systems (SAS) software package (V.9.4; Cary, NC) was used to obtain descriptive statistics on all lung and bronchus cancers as well as SCLC cases by age at diagnosis, sex, race, ethnicity, and cancer stage. Additionally, we assessed trends of SCLC as a proportional of all lung cancers overall and by race and ethnicity from 2000 to 2020. SEER*STAT V.8.4.3 was used to extract all lung cancer data for descriptive statistics and to calculate age‐adjusted incidence rates, annual percentage changes (APC) with 95% confidence intervals using the modified Tiwari et al. method [13], 1‐year observed and relative survival with 95% CIs, and age‐adjusted incidence‐based mortality. All age adjusted rates were standardized to the 2000 U.S. standard population with 19 age groups and are per 100,000 individuals. Lastly, a Mantel–Haenszel test was used to assess the trend in cancer stage proportions by year of diagnosis. Alpha was set to 0.5 for all statistical tests.

## 3 Results

The database included 1,584,746 lung cancer cases, among which 188,426 (11.9%) were SCLC (Figure S1 illustrates proportion of lung cancer based on histology). Most SCLC patients were White (82.6%), 65 years and older (63.3%) and had distant metastatic disease (55.9%) at the time of diagnosis (Table S1). There has been a gradual decline in the proportion of SCLC cases across all the racial and ethnic groups over the past two decades (Table S2 and Figure S2). Non‐Hispanic American Indian/Alaska Native had the highest proportion of SCLC, accounting for 14.4% of all lung cancer cases, albeit with a limited contribution to the sample size. On the other hand, non‐Hispanic Asians/Pacific Islander had the lowest proportion, accounting for 6.7% of all lung cancer cases.

**FIGURE 1:**
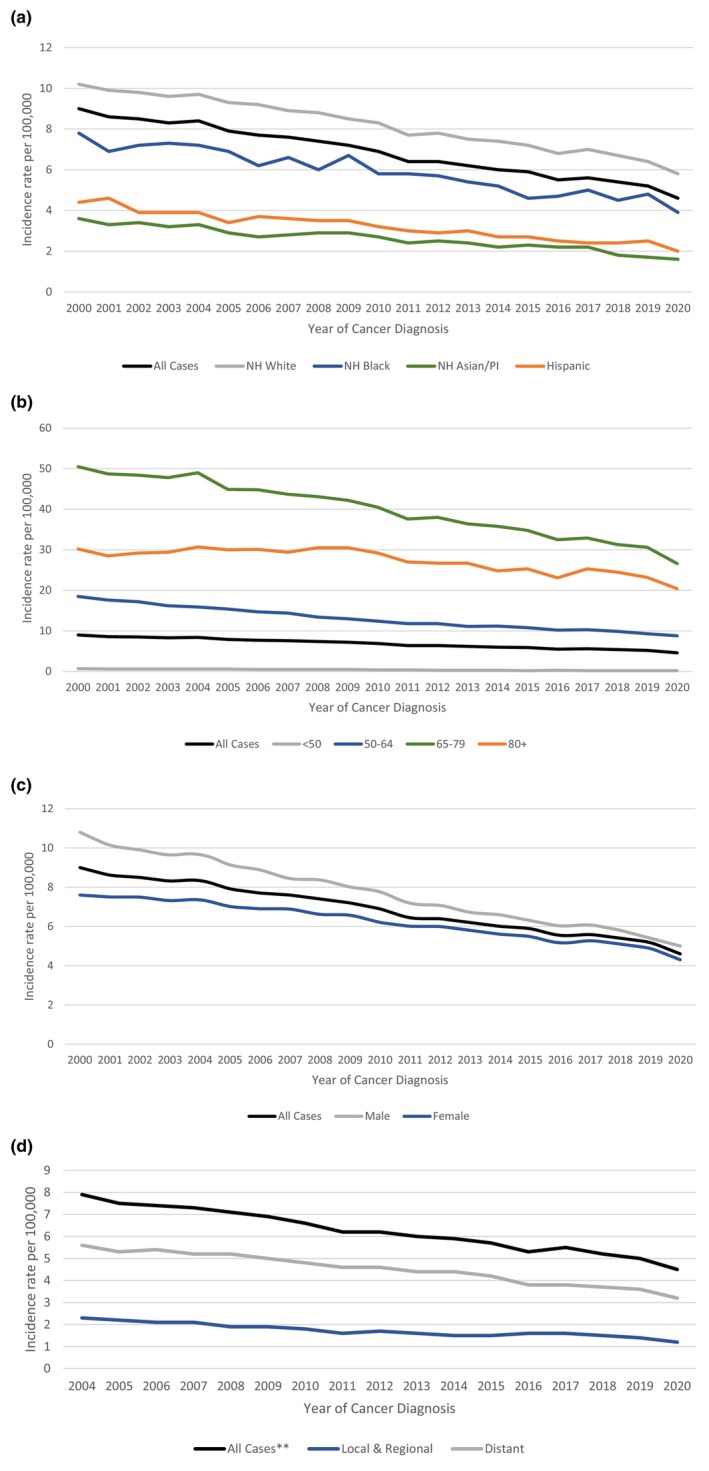
Age‐adjusted incidence rate by (a) ethnic‐racial status, (b) age at diagnosis, (c) sex, and (d) cancer stage. AI/AN, American Indian/Alaska Native, NH, non‐Hispanic; PI, Pacific Islander. **Excluded unknown cancer stage.

The annual age‐adjusted SCLC incidence rate was 9.0 per 100,000 persons in 2000 and decreased to 4.6 per 100,000 in 2020 as shown in Figure 1a. On average, the incidence rate decreased by 3% (95% CI: −3.2% to −2.8%) each year between 2000 and 2020 (Table 1a). This gradual decrease in incidence rates was evident for all races, age groups, sexes, and stages (Figure 1a–d and Tables 1a, 1b, 1c, 1d). NH Asian/Pacific Islanders (APC = −3.5%; 95% CI: −4.0% to −3.1%) and Hispanics (APC = −3.4%; 95% CI: −3.8% to −3.1%), on average, had a significantly higher annual decrease in incidence rates of SCLC compared to NH whites (APC = −2.5%; 95% CI: −2.7% to −2.3%) (Table 1a).

**TABLE 1a:** Percent change (PC) and annual percent change (APC) for age‐adjusted incidence rate by ethnic‐racial status.

	All cases	NH White	NH Black	NH Asian/PI	Hispanic
2000–2020 PC	−48.6	−42.9	−49.8	−55.9	−53.7
2000–2020 APC	−3.0^a^	−2.5^a^	−2.9^a^	−3.5^a^	−3.4^a^
2000–2020 APC 95% CI	(−3.2 to −2.8)	(−2.7 to −2.3)	(−3.3 to −2.5)	(−4.0 to −3.1)	(−3.8 to −3.1)

Abbreviations: AI/AN, American Indian/Alaska Native; APC, annual percent change; CI, confidence interval; NH, non‐Hispanic; PC, percent change; PI, Pacific Islander.

^a^ The APC is significantly different from zero (*p* < 0.05).

**TABLE 1b:** Percent change (PC) and annual percent change (APC) for age‐adjusted incidence rate by age at diagnosis.

	All cases	< 50	50–64	65–79	80+
2000–2020 PC	−48.6	−73.8	−52.3	−47.2	−32.3
2000–2020 APC	−3.0^a^	−6.4^a^	−3.5^a^	−2.8^a^	−1.6^a^
2000–2020 APC 95% CI	(−3.2 to −2.8)	(−7 to −5.7)	(−3.6 to −3.4)	(−3.1 to −2.6)	(−2 to −1.1)

Abbreviations: APC, annual percent change; CI, confidence interval; PC, percent change.

^a^ The APC is significantly different from zero (*p* < 0.05).

**TABLE 1c:** Percent change (PC) and annual percent change (APC) for age‐adjusted incidence rate by sex.

	All cases	Male	Female
2000–2020 PC	−48.6	−53.7	−43.7
2000–2020 APC	−3.0^a^	−3.5^a^	−2.5^a^
2000–2020 APC 95% CI	(−3.2 to −2.8)	(−3.7 to −3.4)	(−2.8 to −2.3)

Abbreviations: APC, annual percent change; CI, confidence interval; PC, percent change.

^a^ The APC is significantly different from zero (*p* < 0.05).

**TABLE 1d:** Percent change (PC) and annual percent change (APC) for age‐adjusted incidence rate by cancer stage.

	All cases^b^	Local and regional	Distant
2004–2020 PC	−43.2	−46.2	−41.9
2004–2020 APC	−3.1^a^	−3.2^a^	−3.1^a^
2004–2020 APC 95% CI	(−3.4 to −2.9)	(−3.8 to −2.7)	(−3.4 to −2.8)

Abbreviations: APC, annual percent change; CI, confidence interval; PC, percent change.

^a^ The APC is significantly different from zero (*p* < 0.05).

^b^ Excluded unknown cancer stage.

There was a small increase in 1‐year relative survival and observed survival between 2000 and 2019 (Table 3 and Table S3). The 1‐year relative survival increased from 33.1% (95% CI: 32.1%–34.2%) among those diagnosed in 2000 to 35.3% (95% CI: 34.1%–36.5%) among those diagnosed in 2019 (Table 3). Likewise, the 1‐year OS was 32.4% (95% CI: 31.3%–33.4%) among those diagnosed in 2000 and 34.5% (95% CI: 33.4%–35.7%) among those diagnosed in 2019 (Table S3). A small but gradual improvement in survival outcomes over the past two decades was evident across most races (Table 3 and Table S3), keeping in mind the relatively smaller size for American Indian/Alaskan native and Asian/Pacific Islander, which could limit the interpretation of their data.

**FIGURE 2:**
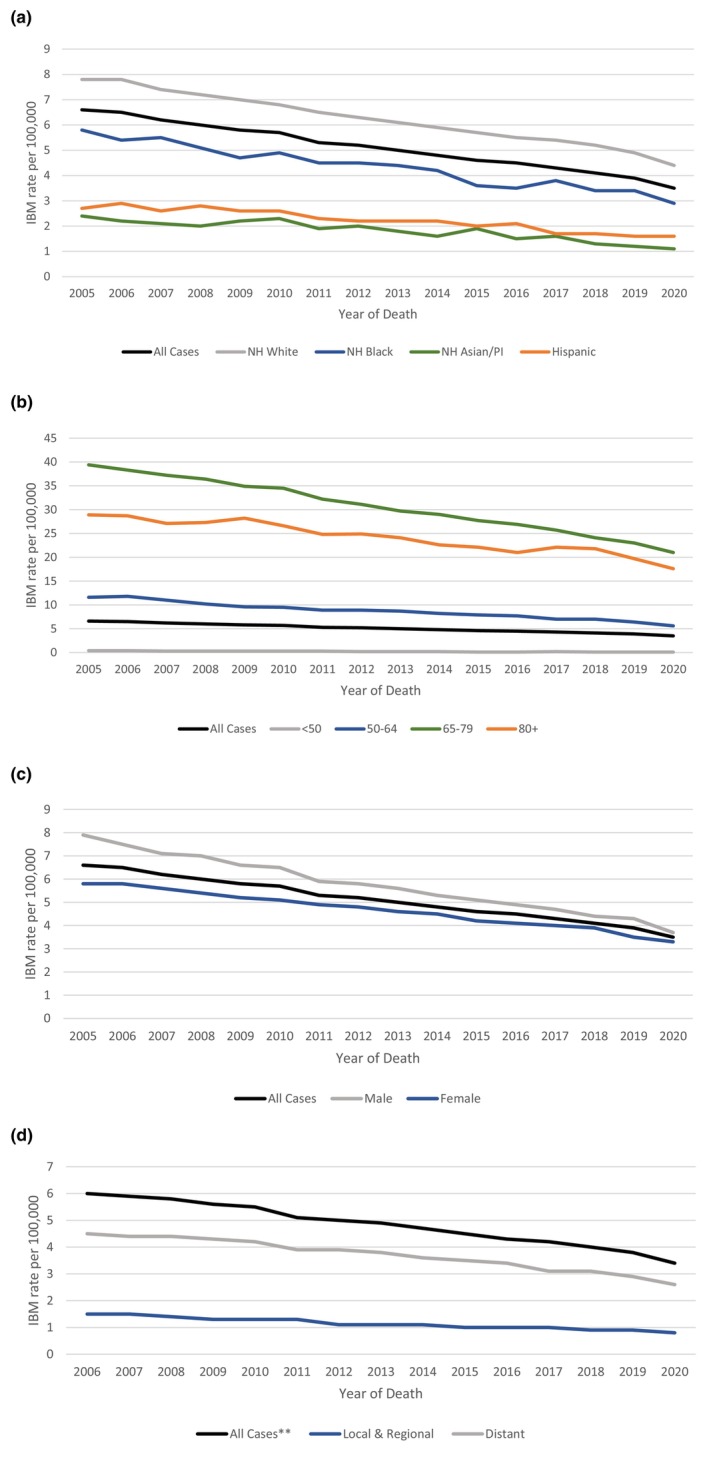
Age‐adjusted lung cancer mortality rate by (a) ethnic‐racial status, (b) age at death, (c) sex, and (d) cancer stage. AI/AN, American Indian/Alaska Native; IBM, incidence‐based mortality; NH, non‐Hispanic; PI, Pacific Islander. **Excluded unknown cancer stage.

The age‐adjusted incidence‐based mortality rate decreased from 6.6 in 2005 to 3.5 in 2020 (Figure 2a and Table 2a). The incidence‐based mortality was highest for non‐Hispanic whites, and for those who were 65 to 79 years of age, while lowest for non‐Hispanic Asian/Pacific Islander and for patients under 50 years of age (Figure 2a,b and Table 2a, 2b). Likewise, males and patients with distant metastatic disease had higher incidence‐based mortality when compared to females (Figure 2c and Table 2c) and patients with local or regional disease, respectively (Figure 2d and Table 2d).

**TABLE 2a:** Percent change (PC) and annual percent change (APC) for age‐adjusted lung cancer mortality rate by ethnic‐racial status.

	All cases	NH White	NH Black	NH Asian/PI	Hispanic
2005–2020 PC	−47.8	−44.1	−50.2	−33.5	−52.9
2005–2020 APC	−4^a^	−3.5^a^	−4^a^	−2.2^a^	−4.4^a^
2005–2020 APC 95% CI	(−4.2 to −3.7)	(−3.7 to −3.2)	(−4.6 to −3.5)	(−3.4 to −0.9)	(−5.5 to −3.2)

Abbreviations: AI/AN, American Indian/Alaska Native; APC, annual percent change; CI, confidence interval; NH, non‐Hispanic; PC, percent change; PI, Pacific Islander.

^a^ The APC is significantly different from zero (*p* < 0.05).

**TABLE 2b:** Percent change (PC) and annual percent change (APC) for age‐adjusted lung cancer mortality rate by age at death.

	All cases	< 50	50–64	65–79	80+
2005–2020 PC	−47.8	−72.9	−51.5	−46.6	−39
2005–2020 APC	−4^a^	−8.4^a^	−4.2^a^	−3.9^a^	−2.9^a^
2005–2020 APC 95% CI	(−4.2 to −3.7)	(−9.4 to −7.4)	(−4.6 to −3.8)	(−4.2 to −3.6)	(−3.3 to −2.4)

Abbreviations: APC, annual percent change; CI, confidence interval; PC, percent change.

^a^ The APC is significantly different from zero (*p* < 0.05).

**TABLE 2c:** Percent change (PC) and annual percent change (APC) for age‐adjusted lung cancer mortality rate by sex.

	All cases	Male	Female
2005–2020 PC	−47.8	−53.3	−42.5
2005–2020 APC	−4^a^	−4.5^a^	−3.5^a^
2005‐2020 APC 95% CI	(−4.2 to −3.7)	(−4.8 to −4.2)	(−3.8 to −3.2)

Abbreviations: APC, annual percent change; CI, confidence interval; PC, percent change.

^a^ The APC is significantly different from zero (*p* < 0.05).

**TABLE 2d:** Percent change (PC) and annual percent change (APC) for age‐adjusted lung cancer mortality rate by cancer stage.

	All cases^b^	Local and regional	Distant
2005–2020 PC	−37.1	−34.1	−38
2005–2020 APC	−3.4^a^	−3.6^a^	−3.3^a^
2005–2020 APC 95% CI	(−3.9 to −2.8)	(−4.5 to −2.7)	(−3.8 to −2.7)

Abbreviations: APC, annual percent change; CI, confidence interval; PC, percent change.

^a^ The APC is significantly different from zero (*p* < 0.05).

^b^ Excluded unknown cancer stage.

**TABLE 3:** One‐year relative survival by ethnic‐racial status.

Year of cancer diagnosis	All cases	NH White	NH Black	NH AI/AN^a^	NH Asian/PI	Hispanic
2000–2019	33.7%	33.5%	35.7%	35.6%	38.1%	32.1%
2000	33.1%	32.9%	35.8%	21.0%	37.8%	32.8%
2001	33.9%	34.0%	32.0%	43.1%	36.0%	33.7%
2002	33.0%	32.9%	33.7%	24.8%	40.6%	30.2%
2003	34.0%	34.1%	33.8%	48.2%	34.3%	30.7%
2004	33.6%	33.7%	32.6%	33.4%	37.7%	30.5%
2005	33.1%	33.1%	32.9%	45.6%	38.1%	30.6%
2006	33.7%	33.6%	36.3%	32.8%	38.6%	30.2%
2007	34.7%	34.0%	36.9%	46.7%	40.5%	36.9%
2008	32.9%	32.9%	33.3%	32.8%	37.0%	29.4%
2009	34.7%	34.7%	34.1%	29.8%	33.1%	35.9%
2010	33.8%	33.5%	36.1%	41.2%	38.8%	31.5%
2011	33.4%	33.1%	35.3%	43.5%	34.0%	33.6%
2012	33.9%	33.2%	40.1%	39.9%	39.0%	31.8%
2013	34.1%	33.2%	38.2%	35.2%	41.8%	34.8%
2014	32.7%	32.7%	36.7%	49.4%	31.9%	26.1%
2015	33.9%	34.0%	34.7%	30.1%	39.4%	29.6%
2016	33.9%	33.6%	37.1%	29.6%	39.6%	31.7%
2017	33.6%	33.2%	34.2%	35.8%	38.1%	34.8%
2018	33.5%	33.0%	38.7%	22.5%	38.7%	32.2%
2019	35.3%	34.5%	38.7%	32.6%	44.9%	35.5%

Abbreviations: AI/AN, American Indian/Alaska Native; NH, non‐Hispanic; PI, Pacific Islander.

^a^ Small sample size.

## 4 Discussion

In this population‐based study, we found that the incidence of SCLC has been declining over the past two decades with a relative decrease of 48.6% between the year 2000 and 2020. Additionally, our study demonstrated that the SCLC now accounts for 11.2% of all lung cancer cases as compared to 13.3% in 2000. A study by Govindan and colleagues similarly demonstrated steady decline in SCLC cases from 17.26% in 1986 to 12.95% in 2002 [1]. Since SCLC almost always occurs in smokers, the decrease in smoking prevalence in the United States, predominately driven by implantation of smoking‐related policies is likely responsible for this notable decline.

The gradual and persistent decrease in the incidence of SCLC was evident across all ages, races/ethnicities, sexes, and stages from 2004 to 2020 (Figure S2). Arrazola and colleagues demonstrated a linear decline in the prevalence of cigarette smoking from 2011 to 2020 among non‐Hispanic White, non‐Hispanic Black, non‐Hispanic Asian, and Hispanic adults [14]. Additionally, a cross‐sectional study utilizing responses to National Health Interview Surveys among 353,555 adults demonstrated a significant decline in smoking prevalence from 2011 to 2022 [15], with more decrease noted among younger adults. Interestingly, our study also noted a pronounced decrease in SCLC incidence in those less than 50 years of age by 73.8%. Our study also demonstrated that the age‐adjusted incidence rate of SCLC is higher in males than in females and is highest among non‐Hispanic whites when compared to non‐Hispanic blacks, Asians and Hispanics. This mirrors the prevalence of smoking across different sexes, as well as different racial and ethnic groups [14, 16]. These findings underscore the importance of smoking avoidance or cessation and anti‐smoking campaigns.

**FIGURE 3:**
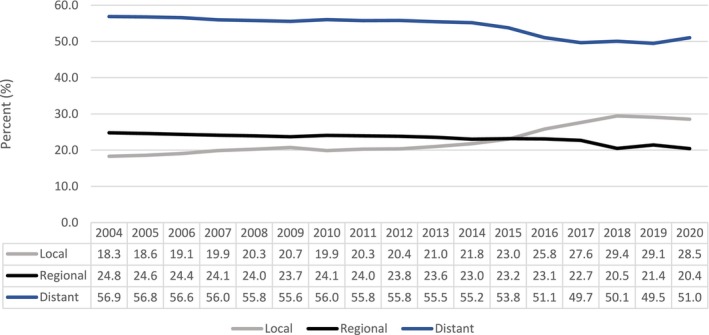
Small cell lung cancer stage proportions by year of diagnosis. Excluded unknown cancer stage.

The United States Preventive Service Task Force recommends an annual screening for lung cancer using low‐dose computed tomography (CT) of the chest for patients between 50 and 80 years of age who have a 20 pack‐year smoking history and who currently smoke or have quit within the past 15 years [17]. This screening guideline was based on the results of the two large randomized clinical trials, namely the United States National Lung Screening Trial and the Dutch–Belgian NEderlands Leuvens Screening ONderzoek (NELSON) trial, which demonstrated reduced mortality in high‐risk patient population with low‐dose CT scan screening [18, 19]. The goal of CT screening is to detect the diseases at earlier stage with the aim for cure. Although CT lung screening has been implemented since 2013 [17], most patients (55.9%) in our study had distant metastatic disease at the time of diagnosis. This suggests that annual CT lung cancer screening may not be an ideal screening tool for SCLC, given the aggressive nature of the disease and the high potential for SCLC to metastasize rapidly within the 1‐year timeframe interval. A study conducted by Thomas and colleagues also demonstrated that when compared to non‐small cell lung cancer, a significantly higher proportion of SCLC cases were diagnosed within 1 year following a negative screening and majority of SCLC cases were diagnosed at late stages [20]. Nonetheless, there is a small notable increase in the incidence of localized disease and a decrease in the incidence of metastatic disease over the years (*p*‐trend < 0.001) (Figure 3). This increase in the diagnosis of localized SCLC became more pronounced after 2013, when lung cancer CT screening became publicly available. The slow trend before 2013 could be reflective of the nationwide increase in the use of diagnostic CT scans for multiple indications. The trend toward higher incidence of localized SCLC, particularly after the implementation of screening CT scan, is encouraging and warrants further research.

Although there was a significant decline in the age‐adjusted incidence‐based mortality rate from 2006 to 2020, our study did not show a significant improvement in survival outcomes. This is likely related to the lack of progress in developing impactful treatment regimen for SCLC over the last two decades. Notably, atezolizumab and durvalumab are two ICIs now approved for use in extensive‐stage SCLC in combination with platinum etoposide. The approvals are based on the results of the two large phase III studies, namely, the IMPOWER‐133 and the CASPIAN study, respectively [4, 5]. Adding these agents resulted in a modest improvement in median OS compared to chemotherapy. Additionally, they have been shown to yield a durable survival benefit in a very small subset of patients. For instance, long‐term observational study of IMPOWER‐133 has shown a 5‐year OS rate of 12% for patients receiving platinum‐etoposide plus atezolizumab [21]. Since atezolizumab was the first ICI approved on March 19, 2019, for extensive‐stage SCLC, given the modest OS benefit and lack of adequate follow‐up duration, our study was not able to capture this OS benefit of ICI in the year 2019 and 2020. We anticipate with longer follow up; we will likely be able to see this survival benefit in SCLC. Nonetheless, there remains a pressing need for more effective systemic therapy for patients with SCLC.

This study has several strengths. Given its large sample size, this is the largest SCLC study to date and includes the most up‐to‐date information on epidemiology and survival outcomes of patients with SCLC at a population level. The SEER database covers approximately 48% of the US population. It also provides comprehensive longitudinal data over 20 years, including demographic and staging statistics in relation to SCLC.

Our study has several limitations. The SEER database is not a national database, which may limit the generalization of our results. Additionally, we were restricted in our assessment of cancer stages to the years 2004–2020, as data before this period was unavailable. Moreover, we used a 5‐year burn‐in period to calculate IBM, meaning IBM trends can only be calculated after the burn‐in period (from 2005 onward), and not for the entire 20 years. Additionally, since IBM data correlate with the year of death and not the year of diagnosis, different trends related to either screening or survival improvements may be highlighted when compared to the incidence data. Finally, the AI/AN population in our study was very small, so only limited conclusions can be drawn about this specific racial population.

In conclusion, our retrospective, population‐based study utilizing the SEER database found that the incidence of SCLC has decreased from 2000 to 2020. This is likely due to a reduction in smoking rates, underscoring the importance of smoking abstinence. These factors, in addition to enhanced medical care, have also resulted in an improvement in incidence‐based mortality. However, the lack of improvement in survival outcomes reflects the need for more effective systemic therapy for patients with SCLC.

## Author Contributions

**Dipesh Uprety:** conceptualization (lead), funding acquisition (lead), investigation (lead), methodology (lead), project administration (lead), supervision (lead), writing – original draft (lead), writing – review and editing (lead). **Randell Seaton:** conceptualization (lead), data curation (lead), formal analysis (lead), investigation (lead), methodology (lead), software (lead), writing – original draft (lead), writing – review and editing (lead). **Abesh Niroula:** writing – original draft (equal), writing – review and editing (equal). **Tarik Hadid:** writing – original draft (equal), writing – review and editing (equal). **Kaushal Parikh:** writing – original draft (equal), writing – review and editing (equal). **Julie J. Ruterbusch:** conceptualization (lead), data curation (lead), formal analysis (lead), investigation (lead), methodology (lead), resources (lead), writing – original draft (lead), writing – review and editing (lead).

## Conflicts of Interest

Advisory Board/ Consulting fees for Daiichi Sankyo, Sanofi, Astrazaneca, and Jazz Pharmaceuticals. Research Consultant: Pfizer; **Randell Seaton:** no conflicts of interest to disclose; **Abesh Niroula:** no conflicts of interest to disclose; **Tarik Hadid:** no conflicts of interest; **Kaushal Parikh**: Research funding from Verastem Oncology to institution. Advisory Board/Consultant for Astrazeneca and Guardant Health (funding to institution). Honoraria from OncLive, MJH Life Sciences and Dava Oncology (to institution); **Julie J Ruterbusch:** no conflicts of interest.

## Supporting information


Data S1.

## Data Availability

This project was completed using the Surveillance, Epidemiology, and End Results (SEER) database. A data use agreement was required to obtain the data. For more information, please visit the following site: https://seer.cancer.gov/data/access.html.
