# Supplementary material for: Trends in the Incidence and Survival Outcomes in Patients With Small Cell Lung Cancer in the United States: An Analysis of the SEER Database
Source: Cancer Med. 2025 Feb 5;14(3):e70608. doi: 10.1002/cam4.70608 (PMC11797299; doi:10.1002/cam4.70608)
Supplement: Supplementary file 1 — Data S1. [file CAM4-14-e70608-s001.docx]

**Supplemental Table 1**: **Characteristics of Lung Cancer Cases Diagnosed Between 2000-2020**

|  | **All Lung & Bronchus** | | **Small Cell Lung Cancer** | |
| --- | --- | --- | --- | --- |
|  | **N** | **%** | **N** | **%** |
| **Total** | 1,584,746 | 100 | 188,426 | 11.9 |
| **Age at Diagnosis (years)** |  |  |  |  |
| <50 | 66,093 | 4.2 | 7,381 | 3.9 |
| 50-64 | 424,611 | 26.8 | 61,752 | 32.8 |
| 65-79 | 776,633 | 49.0 | 95,566 | 50.7 |
| 80+ | 317,409 | 20.0 | 23,727 | 12.6 |
| **Sex** |  |  |  |  |
| Male | 837,969 | 52.9 | 94,790 | 50.3 |
| Female | 746,777 | 47.1 | 93,636 | 49.7 |
| **Ethnic-racial group** |  |  |  |  |
| NH White | 1,224,578 | 77.3 | 155,603 | 82.6 |
| NH Black | 165,547 | 10.5 | 15,408 | 8.2 |
| NH AI/AN | 6,466 | 0.4 | 934 | 0.5 |
| NH Asian/PI | 76,895 | 4.9 | 5,116 | 2.7 |
| Hispanic (all races) | 109,293 | 6.9 | 11,231 | 6.0 |
| NH Unknown Race | 1,967 | 0.1 | 134 | 0.1 |
| **Cancer Stage (2004+)** |  |  |  |  |
| Local | 269,316 | 17.0 | 8,373 | 4.4 |
| Regional | 276,491 | 17.5 | 31,856 | 16.9 |
| Distant | 643,871 | 40.6 | 105,336 | 55.9 |
| Unknown/unstaged | 105,392 | 6.7 | 5,406 | 2.9 |

Abbreviations: NH – Non-Hispanic, AIAN – American Indian/Alaska Native, PI – Pacific Islander

**Supplemental Table 2**: **Small Cell Lung Cancer as a Proportion of All Lung Cancers by Ethnic-racial Status**

| **Year of Cancer Diagnosis** | **All Cases** | **NH White** | **NH Black** | **NH AI/AN*** | **NH Asian/PI** | **Hispanic** |
| --- | --- | --- | --- | --- | --- | --- |
| 2000-2020 | 11.9 | 12.7 | 9.3 | 14.4 | 6.7 | 10.3 |
| 2000 | 13.3 | 13.9 | 10.3 | 18.2 | 8.7 | 12.5 |
| 2001 | 12.9 | 13.5 | 9.2 | 15.3 | 8.3 | 12.7 |
| 2002 | 12.9 | 13.5 | 10.1 | 16.5 | 8.6 | 11.3 |
| 2003 | 12.7 | 13.3 | 10.0 | 17.3 | 8.0 | 11.2 |
| 2004 | 13.0 | 13.8 | 10.2 | 19.6 | 8.0 | 11.1 |
| 2005 | 12.4 | 13.1 | 9.6 | 16.3 | 7.3 | 10.1 |
| 2006 | 12.3 | 13.1 | 9.0 | 11.7 | 6.8 | 11.2 |
| 2007 | 12.1 | 12.8 | 9.7 | 15.1 | 7.1 | 10.6 |
| 2008 | 12.0 | 12.8 | 8.9 | 10.8 | 7.5 | 10.8 |
| 2009 | 12.0 | 12.7 | 9.9 | 15.2 | 7.4 | 11.2 |
| 2010 | 12.0 | 12.8 | 9.1 | 18.4 | 7.0 | 10.4 |
| 2011 | 11.5 | 12.2 | 9.4 | 12.1 | 6.4 | 9.9 |
| 2012 | 11.7 | 12.5 | 9.1 | 16.8 | 6.8 | 10.1 |
| 2013 | 11.5 | 12.3 | 9.4 | 11.9 | 6.3 | 10.0 |
| 2014 | 11.5 | 12.4 | 9.0 | 9.7 | 6.1 | 9.7 |
| 2015 | 11.3 | 12.2 | 8.4 | 11.8 | 6.4 | 10.0 |
| 2016 | 10.9 | 11.7 | 8.6 | 11.9 | 6.1 | 9.0 |
| 2017 | 11.2 | 12.1 | 9.3 | 12.5 | 5.9 | 8.9 |
| 2018 | 11.1 | 12.2 | 8.6 | 19.2 | 5.2 | 9.0 |
| 2019 | 10.9 | 11.8 | 9.2 | 14.1 | 4.9 | 9.6 |
| 2020 | 11.2 | 12.2 | 8.9 | 14.1 | 5.7 | 9.5 |
| **Relative % change (2000 to 2020):** | -16% | -12% | -14% | -23% | -34% | -24% |
| Abbreviations: NH – Non-Hispanic, AI/AN – American Indian/Alaska Native, PI – Pacific Islander  *Small sample size. | | | | | | |

**Supplemental Table 3: 1-Year Overall Survival by Ethnic-Racial Status**

| **Year of Cancer Diagnosis** | **All Cases** | **NH White** | **NH Black** | **NH AI/AN*** | **NH Asian/PI** | **Hispanic** |
| --- | --- | --- | --- | --- | --- | --- |
| 2000-2019 | 33.0% | 32.8% | 34.8% | 34.7% | 37.5% | 31.5% |
| 2000 | 32.4% | 32.1% | 34.7% | 20.6% | 37.1% | 32.1% |
| 2001 | 33.1% | 33.2% | 31.1% | 41.9% | 35.3% | 33.0% |
| 2002 | 32.3% | 32.2% | 32.8% | 24.2% | 39.8% | 29.6% |
| 2003 | 33.2% | 33.3% | 32.9% | 47.2% | 33.7% | 30.2% |
| 2004 | 32.8% | 32.9% | 31.8% | 32.6% | 37.1% | 29.9% |
| 2005 | 32.4% | 32.4% | 32.1% | 44.7% | 37.5% | 30.1% |
| 2006 | 33.0% | 32.8% | 35.4% | 32.1% | 37.9% | 29.7% |
| 2007 | 33.9% | 33.3% | 36.0% | 45.7% | 39.8% | 36.2% |
| 2008 | 32.2% | 32.3% | 32.5% | 32.1% | 36.3% | 28.9% |
| 2009 | 34.0% | 33.9% | 33.4% | 29.4% | 32.6% | 35.3% |
| 2010 | 33.2% | 32.9% | 35.3% | 40.4% | 38.2% | 31.0% |
| 2011 | 32.7% | 32.4% | 34.6% | 42.4% | 33.5% | 33.0% |
| 2012 | 33.3% | 32.5% | 39.2% | 38.8% | 38.5% | 31.3% |
| 2013 | 33.4% | 32.6% | 37.4% | 34.2% | 41.2% | 34.2% |
| 2014 | 32.0% | 32.0% | 35.8% | 48.4% | 31.5% | 25.7% |
| 2015 | 33.2% | 33.3% | 33.9% | 29.4% | 38.8% | 29.1% |
| 2016 | 33.2% | 32.9% | 36.3% | 28.9% | 39.0% | 31.2% |
| 2017 | 32.9% | 32.5% | 33.4% | 35.0% | 37.6% | 34.2% |
| 2018 | 32.8% | 32.3% | 37.8% | 21.9% | 38.1% | 31.6% |
| 2019 | 34.5% | 33.7% | 37.7% | 31.7% | 44.3% | 34.7% |
| Abbreviations: NH – Non-Hispanic, AI/AN – American Indian/Alaska Native, PI – Pacific Islander | | | | | | |

*Small sample size.

**Supplementary Figure 1: Proportion of different lung cancer subtypes based on the year of diagnosis**

**Supplemental Figure 2a:** **Natural Logarithm of** **Age-adjusted Incidence Rate by Ethnic-racial Status**

**Supplemental Figure 2b:** **Natural Logarithm of** **Age-adjusted Incidence Rate by Age at Diagnosis**

**Supplemental Figure 2c: Natural Logarithm of Age-adjusted Incidence Rate by Sex**

**Supplemental Figure 2d:** **Natural Logarithm of Age-adjusted Incidence Rate by Cancer Stage**

**Excluded unknown cancer stage.

**Supplemental Figure 3a: Natural Logarithm of Age-adjusted Lung Cancer Mortality Rate by Ethnic-racial Status**

**Supplemental Figure 3b:** **Natural Logarithm of Age-adjusted Lung Cancer Mortality Rate by Age at Death**

**Supplemental Figure 3c:** **Natural Logarithm of Age-adjusted Lung Cancer Mortality Rate by Sex**

**Supplemental Figure 3d:** **Natural Logarithm of Age-adjusted Lung Cancer Mortality Rate by Cancer Stage**

**Excluded unknown cancer stage.
